# Thermodynamic Behavior of (2-Propanol + 1,8-Cineole) Mixtures: Isothermal Vapor–Liquid Equilibria, Densities, Enthalpies of Mixing, and Modeling

**DOI:** 10.3390/ijms241210380

**Published:** 2023-06-20

**Authors:** Beatriz Gimeno, Santiago Martinez, Ana M. Mainar, José S. Urieta, Pascual Perez

**Affiliations:** 1 Departamento de Química Física, Facultad de Ciencias, Universidad de Zaragoza, 50009 Zaragoza, Spain; beatrizgimeno@britanico-aragon.edu (B.G.); yagomasip@gmail.com (S.M.); ammainar@unizar.es (A.M.M.); 2 Group of Applied Thermodynamics and Surfaces (GATHERS), Aragon Institute for Engineering Research (I3A), 50018 Zaragoza, Spain; 3 Instituto Agroalimentario de Aragón (I2A), Centro de Investigación y Tecnología Agroalimentaria de Aragón (CITA), Universidad de Zaragoza, 50009 Zaragoza, Spain

**Keywords:** 1,8-cineole, 2-propanol, vapor pressure, phase equilibria, excess thermodynamic functions, PRM, PRSV EoS, volume translation, SAFT

## Abstract

Vapor pressures and other thermodynamic properties of liquids, such as density and enthalpy of mixtures, are the key parameters in chemical engineering for designing new process units, and are also essential for understanding the physical chemistry, macroscopic and molecular behavior of fluid systems. In this work, vapor pressures between 278.15 and 323.15 K, densities and enthalpies of mixtures between 288.15 and 318.15 K for the binary mixture (2-propanol + 1,8-cineole) have been measured. From the vapor pressure data, activity coefficients and excess Gibbs energies were calculated via the Barker’s method and the Wilson equation. Excess molar volumes and excess molar enthalpies were also obtained from the density and calorimetric measurements. Thermodynamic consistency test between excess molar Gibbs energies and excess molar enthalpies has been carried out using the Gibbs–Helmholtz equation. Robinson–Mathias, and Peng–Robinson–Stryjek–Vera together with volume translation of Peneloux equations of state (EoS) are considered, as well as the statistical associating fluid theory that offers a molecular vision quite suitable for systems having highly non-spherical or associated molecules. Of these three models, the first two fit the experimental vapor pressure results quite adequately; in contrast, only the last one approaches the volumetric behavior of the system. A brief comparison of the thermodynamic excess molar functions for binary mixtures of short-chain alcohol + 1,8-cineole (cyclic ether), or +di-n-propylether (lineal ether) is also included.

## 1. Introduction

Vapor pressures and other thermodynamic properties of pure liquid and mixtures are physical properties of great importance for not only being necessary for the daily challenges in chemical engineering but also from a theoretical point of view as they help to develop and improve representative models of the liquid state. 

Within this context, the mixture (2-propanol + 1,8-cineole) is of particular interest due to the nature of the molecules it comprises, and because of its potential applications, as described below. In this work, an experimental study of vapor–liquid equilibria (VLE), densities, and enthalpies of mixing for the mixture (2-propanol + 1,8-cineole), together with some current models to fit them, are addressed.

1,3,3-Trimethyl-2-oxabicyclo[2.2.2]octane is a cyclic ether usually known as 1,8-cineole, or more commonly as eucalyptol because it is the main component of essential oil obtained from the eucalyptus, although it can be identified in the extracts of many others plants. The fairly recent paper of Cai et al. [1] includes a detailed review on sources, biological activities, and applications of this compound. 1,8-cineole is frequently used in food [2,3,4], cosmetics and care products [4,5], and has been used profusely in pharmaceutical trial [1,4]. It is effective as a mucolytic agent in inflammatory airway medical conditions, such as chronic obstructive pulmonary disease [6] and asthma [7]. Additionally, it has been claimed for many other biological properties, such as antimicrobial [5,8,9], antifungal [10], anxiolytic [11], and it has even been related to skin penetration enhancement of drugs [12]. More recently, other studies have been carried out on the biological activity of 1,8-cineole for the prevention of depressive-like behaviors [13], battle against ovarian and lung cancers [14,15], treatment of Alzheimer’s disease [16,17] and the treatment of conditions, such as co-infections associated with SARS-CoV-2 (COVID-19) [18], among others.

Moreover, 1,8-cineole could be considered an environmentally friendly solvent [19,20] as it behaves as a low toxicity compound [21], and it is obtained mainly from renewable sources [1]. Regarding the extraction process of 1,8-cineole from vegetal material, one of the possible ways is to use supercritical CO_2_ [22], and taking into account the low polarity of CO_2_, it is convenient to add a polarity modifier in order to increase the yield of extraction. Usually, short-chain alcohols are used for that goal. Consequently, VLE and, in general, thermodynamic behavior of 2-propanol + 1,8-cineole mixture could be an interesting guide for the 1,8-cineole separation processes, even if conditions of extraction are somewhat different from those considered here.

Propan-2-ol, the simplest secondary alcohol, also called 2-propanol, isopropanol or isopropyl alcohol, is a well-known compound because of its use in the manufacturing of a wide variety of chemicals and products, and also due to its potential to be used as a multipurpose green solvent. In fact, although 2-propanol is currently obtained via synthesis (through hydration reaction of propene or by hydrogenating acetone), it appears among the first positions in the guides for the greenest solvents recommended according to safety, occupational health and environmental criteria [23]. Additionally, a sustainable synthesis of propanol from renewable glycerol has been proposed [24] in order to not only deliver opportunities to the biodiesel industry, but also to develop a green production route from the biomass-derived glycerol.

Another interesting feature in relation to their possible use as additives for diesel or gasoline is that not only bio-cineole [25] and 2-propanol [26] have been separately considered, but also types of ether/alcohol mixtures [27] such as the system analyzed in this work.

On the other hand, from a theoretical point of view, alcohol + ether mixtures are of great interest because ether molecules could compete with alcohol molecules in hydrogen bond formation through the lone electronic pairs of oxygen atom of ether [28,29].

In recent years, some papers have been published on the thermodynamic properties of both pure 1,8-cineole [30,31] and its mixtures with alcohols [32,33,34,35,36,37]. However, as far as we know, until the present work, no data on vapor pressure, *P*, excess molar enthalpies, $H_{m}^{E}$ , and density, *ρ*, at the considered temperatures of this binary system could be found in the literature. Additionally, in this work, the activity coefficients, *γ_i_*, and the excess molar Gibbs energies, $G_{m}^{E}$ , were determined from experimental *P* data.

Our results on *P* are correlated by three equations of state (EoS): two of them are modifications of the Peng–Robinson (P-R) EoS [38], and a third equation is based on the statistical associating fluid theory (SAFT) [39].

EoS are useful approaches to describe the thermodynamic properties of fluid systems and phase equilibria. Since van der Waal, on the basis of the attractive forces between molecules and their spatial volumes, built his groundbreaking cubic equation [40], hundreds of EoS have been proposed along the years [41,42,43].

Peng and Robinson introduced a modification [38] of the attractive term of such equations in order to provide a more adequate prediction for densities of liquids. In this work, this modification is considered together with that proposed by Mathias [44] to improve the results when considering systems with polar substances, by the introduction of a polar parameter in the temperature depending on the attractive term of the EoS. On the other hand, the Stryjek and Vera modification [45] to the Peng–Robinson equation is considered. In this version, an adjustable parameter for pure components is introduced together with a modification of the polynomial for the acentric parameters.

In short, the EoS of Peng–Robinson–Mathias [44], PRM, and Peng–Robinson–Stryjek–Vera [45], PRSV, together with volume translation (VT) according to Peneloux [46] for both models are considered in this work.

Finally, as previously indicated, experimental results are compared with that of SAFT. The development and success of this model was due to the progress in the statistical mechanics methods, especially the perturbation theory and the valuable contribution of Wertheim [47,48] for chain and association effects. It provided a reasonably simple and accurate [49] general formulation which is accepted as the basis of the SAFT free energy model. This theoretical approach was derived by Chapman et al. [50,51], resulting in a well-grounded EoS, especially in the case of chain-like molecules and when effect of association is remarkable. In fact, the model may simulate the behavior of a wide range of systems having molecules ranging from nearly spherical non-associating to non-spherical associated ones, passing through the intermediate structural configurations. This third situation becomes the case of the system considered in this work, for which molecules are not very far from a spherical shape and one of the components is markedly associated while the other is not. Thus, for our intermediate system, it is interesting to compare the results of this particular SAFT molecular association model with those of the Van der Waals Modified Cubic EoS PRM, and PRSV previously mentioned. A number of user-friendly reviews on the derivation and applications of the SAFT model and the cubic EoS can be found in the literature [39,52,53,54,55,56,57].

It has been for the purpose of enhancing the vision of the models and the molecular interactions, that in addition to the measurements of *P*, the experimental determination of *ρ* and heats of mixture were carried out. In fact, the corresponding thermodynamic properties, $V_{m}^{E}$ , and $H_{m}^{E}$ , are very sensitive to the spatial effects derived from molecular geometry and the detail of intermolecular forces, which complements information provided by other thermophysical quantities [35,58,59]. Furthermore, the present work is also a part of a comprehensive study conducted by our group using 1.8-cineole as cyclic ether and a short-chain alcohol as the second component [32,33,34,35,36,37,60,61]. Likewise, in previous years, we also carried out a similar study with di-n-propylether as linear ether, and an alcohol as the second component of a series that also included the same short-chain alcohols [62,63]. Hence, we include a brief comparison of the different thermodynamic behavior of both sets of systems in the Discussion section.

## 2. Results and Discussion

### 2.1. Pure Components

The molar volumes of the pure components, *V*^0^, used in the Barker analysis, together with the experimental vapor pressures, *P*^0^, which are compared with the values in the literature are gathered in Table 1.

Experimental *P*^0^ data of 2-propanol at ten temperatures between (278.15 K and 323.15 K) were fitted to an Antoine equation (Equation (1)):

(1) $$\ln\left(P^{0}/\mathrm{kPa}\right)=17.6939-\left[4114.55/\left(T/K-39.969\right)\right]$$

The standard deviation of the experimental pressures with respect to that calculated are obtained according to Equation (2):

(2) $$s={\left\{\sum_{i=1}^{N}{\left(X-X_{calc}\right)}_{i}^{2}/\left(N-n\right)\right\}}^{1/2}$$

**Figure 1:**
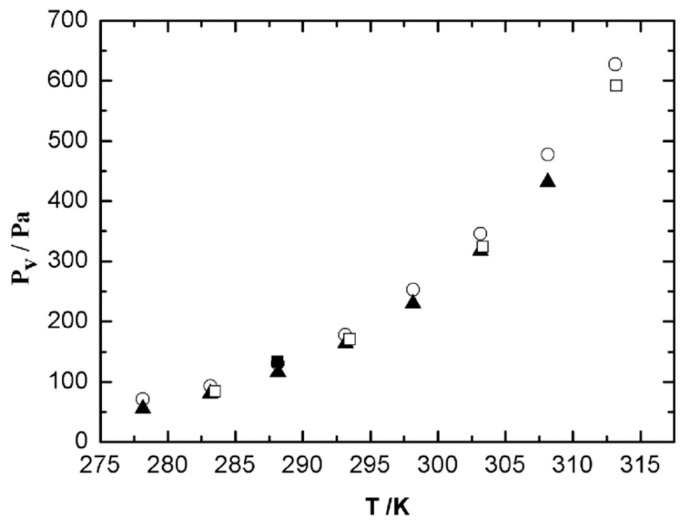
Vapor pressure of pure 1,8-cineole in the temperature range 278.15 K–313.15 K. ○, this work; ■, Stull et al. [66]; ▲, Štejfa et al. [31]; and □, Guetachew et al. [67].

*X* and *X_calc_* correspond to the experimental pressures and calculated values, respectively, *N* is the number of experimental data and n is the number of adjusted parameters. Standard deviation results in a value of 25 Pa, 15 Pa being the maximum deviation at 298.15 K. The corresponding Antoine equation for 1,8-cineole was published previously [36]. Some new experimental vapor pressure data of this liquid have been published in recent years and they are shown in Figure 1 along with our data. The interval of temperature selected in Figure 1 allows for a better comparison between the data from different authors.

The second virial coefficient at *T* = 325.0 K of 2-propanol (*B*_11_ = −1810 × 10^6^ m^3^·mol^−1^ and 1,8-cineole (*B*_22_ = −5490 × 10^6^ m^3^·mol^−1^) were calculated from the Tsonopoulos correlation [68]. For mixtures, the cross virial coefficient, *B*_12_, will be calculated according to the cubic Lorenz semiempirical combination rule [69], as mentioned in Equation (3),

(3) $$B_{12}=\frac{1}{8}{\left(B_{11}^{1/3}+B_{22}^{1/3}\right)}^{3}$$

### 2.2. Vapor Pressures and Derived Thermodynamic Parameters

Table 2 shows our vapor pressure measurements along with the activity coefficients, *γ*_1_ and *γ*_2_, and the excess molar Gibbs free energy, $G_{m}^{E}$ , values fitted using Barker’s method [70] to the Wilson correlation [71].

(4) $$\frac{G_{m}^{E}}{RT}=-x_{1}\ln\left(x_{1}+\Lambda_{12}x_{2}\right)-x_{2}\ln\left(x_{2}+\Lambda_{21}x_{1}\right)$$

The activity coefficients are obtained through the appropriate differentiation of Equation (4).

(5) $$\ln\gamma_{1}=-\ln(x_{1}+\Lambda_{12}x_{2})+x_{2}\left[\frac{\Lambda_{12}}{x_{1}+\Lambda_{12}x_{2}}-\frac{\Lambda_{21}}{\Lambda_{21}x_{1}+x_{2}}\right]$$

(6) $$\ln\gamma_{2}=-\ln(x_{2}+\Lambda_{21}x_{1})-x_{1}\left[\frac{\Lambda_{12}}{x_{1}+\Lambda_{12}x_{2}}-\frac{\Lambda_{21}}{\Lambda_{21}x_{1}+x_{2}}\right]$$

with:

(7) $$\Lambda_{\mathrm{ij}}=\frac{V_{j}^{0}}{V_{i}^{0}}\exp\left(-\frac{\lambda_{ij}-\lambda_{ii}}{RT}\right)$$

where the subscripts 1 and 2 stand for 2-propanol and 1,8-cineole, respectively. *V*^0^ is the molar volume and *λ*’s are the interaction constants between the molecules designated in the subscripts.

The vapor pressure is then given by,

(8) $$P_{\mathrm{calc}}=x_{1}\gamma_{1}P_{1}^{o}R_{1}+x_{2}\gamma_{2}P_{2}^{o}R_{2},$$

and the non-ideality of the vapor phase is taken into account with the following corrections (Equations (9) and (10)):

(9) $$R_{1}=\exp\left\{\left[\left(V_{1}^{o}-B_{11}\right)\left(P-P_{1}^{o}\right)-P\delta_{12}y_{2}^{2}\right]/RT\right\}$$

(10) $$R_{2}=\exp\left\{\left[\left(V_{2}^{o}-B_{22}\right)\left(P-P_{2}^{o}\right)-P\delta_{12}y_{1}^{2}\right]/RT\right\}$$

where $y_{1}$ and $y_{2}$ are the vapor phase mole fractions of 1-propanol and 1,8-cineole, respectively and $\delta_{12}$ is defined by the following equation:

(11) $$\delta_{12}=2B_{12}-B_{11}-B_{22}$$

For every liquid mixture, the vapor pressure is measured at different temperatures from 278.15 to 323.15 K, so a slight modification of the true initial liquid mole fraction can be detected in Table 2, because of the variation in the amount and composition of the vapor phase.

In Table 3, the Wilson parameters, *Λ*_12_ and *Λ*_21_, are collected, together with the standard deviations, defined by Equation (2).

In parentheses, the values mentioned are those that linearly fitted.

**Figure 2:**
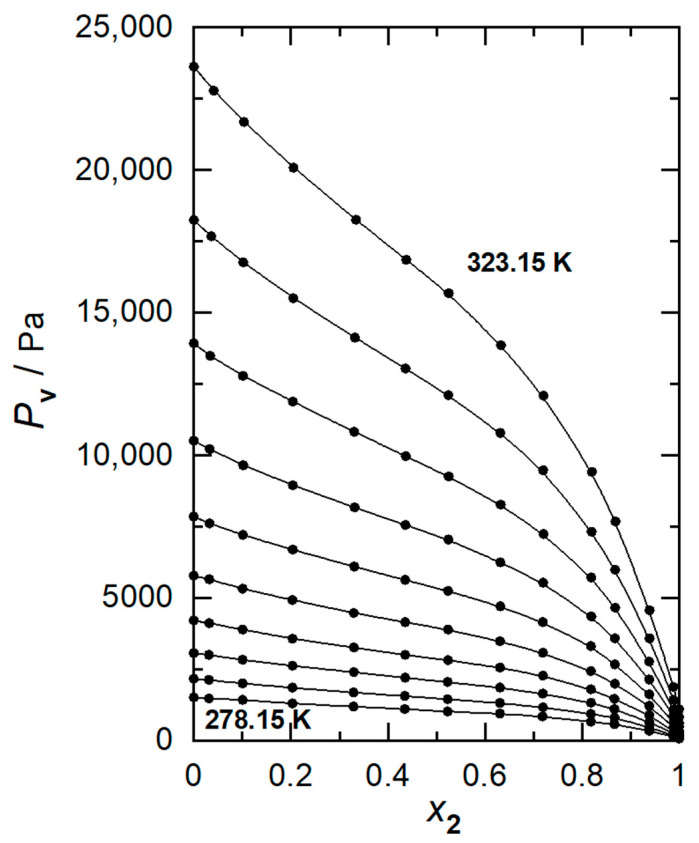
Vapor pressures plotted against liquid-phase composition of 1,8-cineole, at working temperatures between 278.15 K and 323.15 K at intervals of 5 K, for 2-propanol (1) + 1,8-cineole (2)): (●) experimental values, (—) polynomial curve fitting.

**Figure 3:**
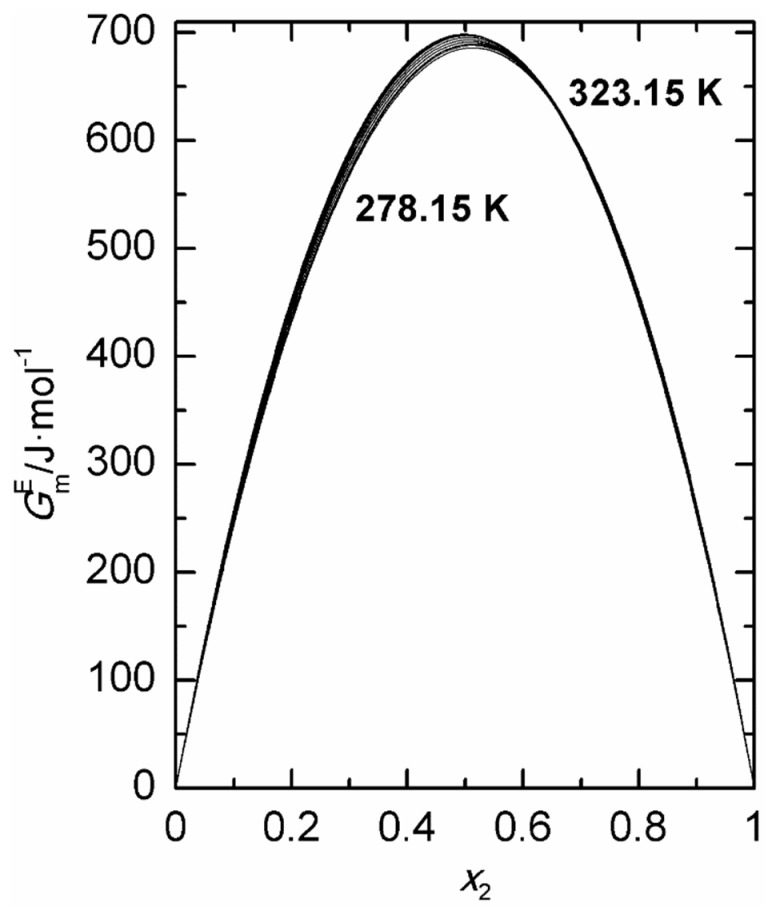
Excess molar Gibbs energies, $G_{m}^{E}$ , calculated via the Barker’s method, at temperatures between 278.15 K and 323.15 K at intervals of 5 K, for 2-propanol (1) + 1,8-cineole (2), plotted as a function of mole fraction of 1,8-cineole.

In the same table, Wilson coefficients, *λ*_12_ − *λ*_11_ and *λ*_12_ − *λ*_22_, obtained from Equation (7), as well as values linearly fitted with temperature are also presented. Vapor pressure–liquid composition curves are shown in Figure 2. On the other hand, Figure 3 shows graphically the analytic calculations for $G_{m}^{E}$ .

The negatively defined Wilson coefficients, *λ*_ij_, stand for energies of interaction between the molecules type i and j. By combining our previous *λ*_ij_ values published for the 2-propanol (1) + di-n-propylether (2) system [62] and the *λ*_ij_ values for the 2-propanol (1) + 1,8-cineole (2) system from Table 3 at *T* = 298.15 K, we can obtain the differences:

$$\begin{array}{c} \lambda_{12}-\lambda_{12^{\prime }}=851\;J\cdot {\mathrm{mol}}^{-1}\\ \lambda_{22}-\lambda_{2^{\prime }2^{\prime }}=480\;J\cdot {\mathrm{mol}}^{-1} \end{array}$$

where the notation used is 1 for 2-propanol, 2 for di-n-propylether and 2′ for 1,8-cineole.

These differences point to a stronger energy of interaction of 2-propanol-1,8-cineole than 2-propanol-di-n-propylether and also a stronger energy of interaction of 1,8-cineole-1,8-cineole than di-n-propylether-di-n-propylether, taking into account that the calculated numerical values are relative and also the approximate nature of the Wilson model.

### 2.3. Excess Molar Enthalpies and Densities

Experimental excess molar enthalpies and densities at four temperatures are gathered in Table 4 and Table 5, respectively.

**Figure 4:**
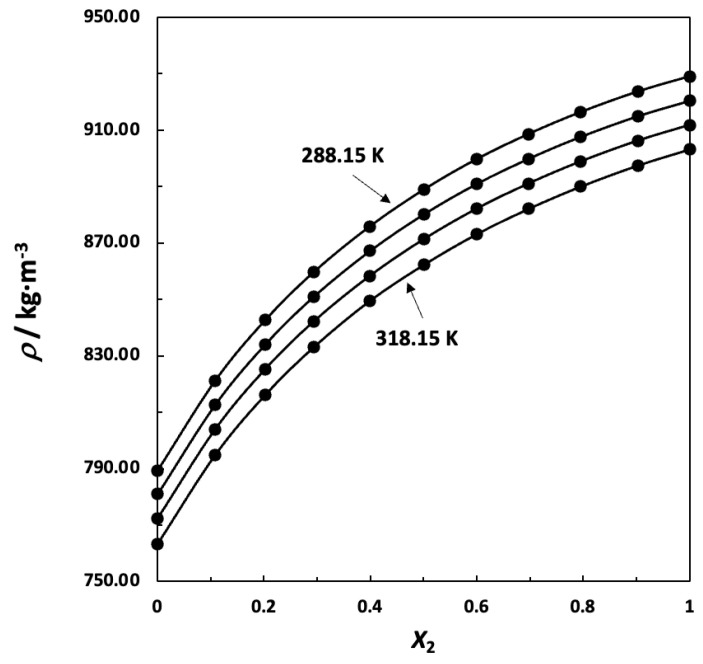
Densities, *ρ*, of mixtures 2-propanol (1) + 1,8-cineole (2) at four temperatures (288.15, 298.15, 308.15 and 318.15 K): (●) experimental values, (—) polynomial curve fitting.

Graphical representations of experimental densities as function of composition at the four temperatures considered appears in Figure 4.

Excess molar volumes were calculated using Equation (11):

(12) $$V_{m}^{E}=x_{1}M_{1}\left(\frac{1}{\rho }-\frac{1}{\rho_{1}}\right)+x_{2}M_{2}\left(\frac{1}{\rho }-\frac{1}{\rho_{2}}\right)$$

where 𝜌 stands for experimental density of the mixture and subscripts 1 and 2 for 2-propanol and 1,8-cineole, respectively.

The values of the excess molar properties, $H_{m}^{E}$ and $V_{m}^{E}$ , have been fitted via least squares to a Redlich–Kister polynomial:

(13) $$Q_{m}^{E}=x_{1}x_{2}\sum_{j=1}^{k}A_{j}{\left(x_{1}-x_{2}\right)}^{j}$$

where $Q_{m}^{E}$ denotes $H_{m}^{E}$ or $V_{m}^{E}$ , *x*_1_ and *x*_2_ represent the mole fraction of 2-propanol and 1,8-cineole, respectively.

The coefficients of Equation (13) are collected in Table 6 beside the standard deviation $s\left(Q_{m}^{E}\right)$ obtained from Equation (2).

**Figure 5:**
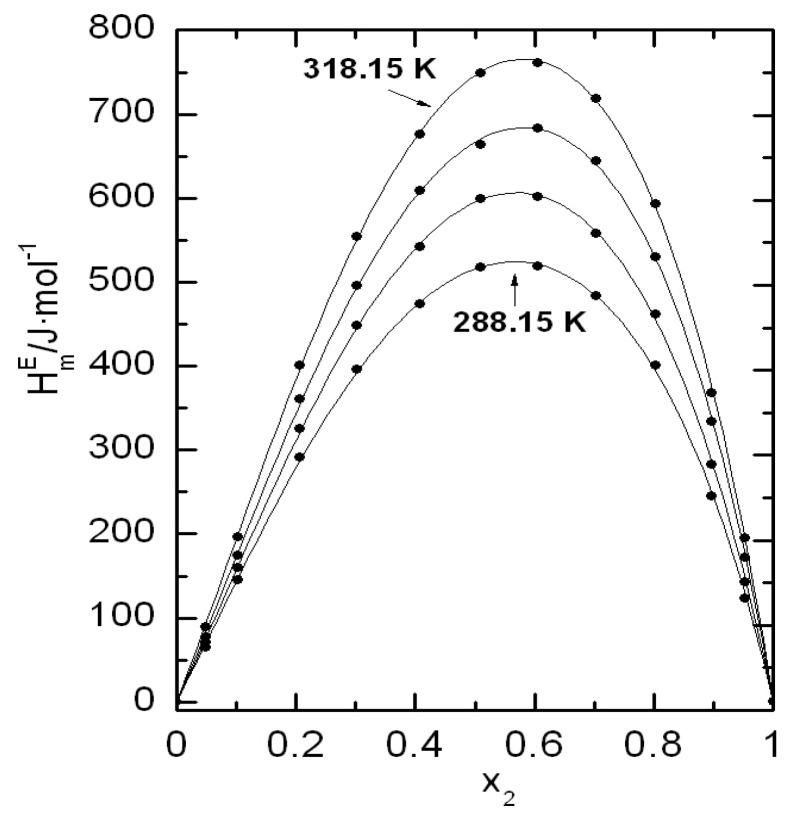
Excess molar enthalpies, $\;H_{m}^{E}$ , at four temperatures for 2-propanol (1) + 1,8-cineole (2): (●) experimental values; (—) Equation (12).

**Figure 6:**
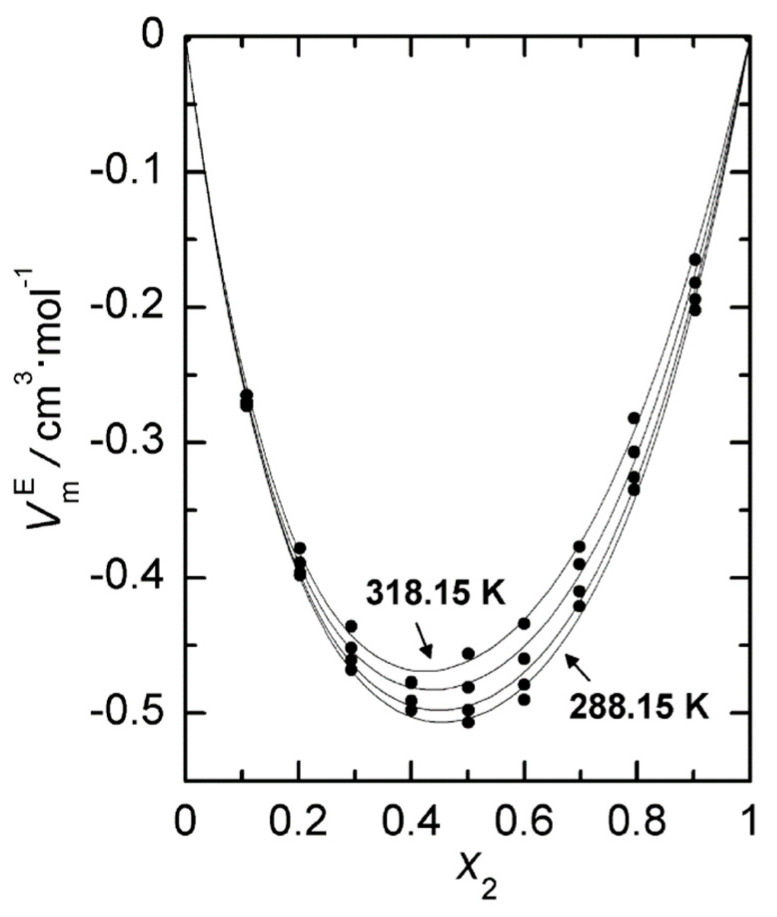
Excess molar volumes, $\;V_{m}^{E}$ , at four temperatures for 2-propanol (1) + 1,8-cineole (2): (●) experimental values; (—), Equation (12).

Graphical representations of both excess molar properties, $\;H_{m}^{E}$ and $\;V_{m}^{E}$ , are plotted in Figure 5 and Figure 6, respectively. Both excess molar properties increase with temperature and in the case of excess molar enthalpies that increase at molar fraction around 0.5 is almost lineal, so we can calculate a value of $C_{P,m}^{E}\approx 7.6\;J\cdot {\mathrm{mol}}^{-1}K^{-1}$ .

**Figure 7:**
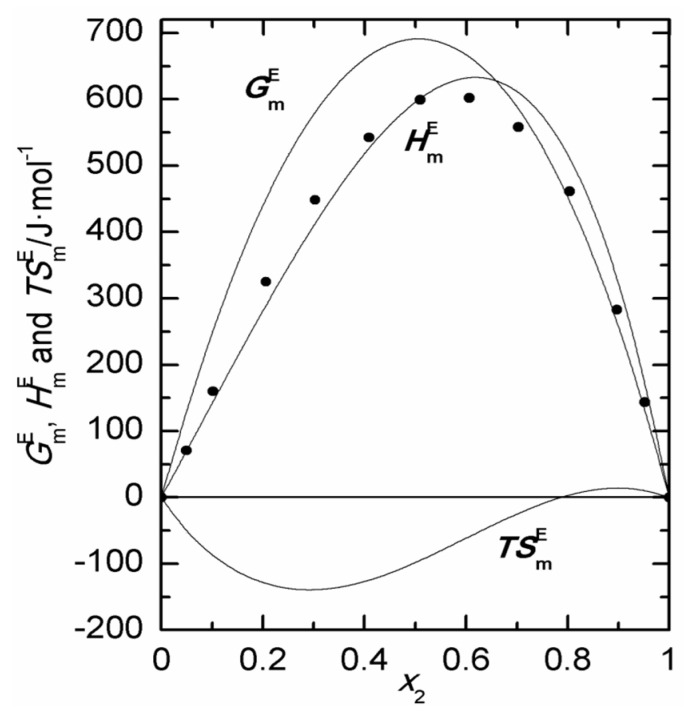
Thermal excess molar functions at 298.15 K for 2-propanol (1) + 1,8-cineole (2): (●) experimental $H_{m}^{E}$ ; (—) Gibbs–Helmholtz $H_{m}^{E}$ , $G_{m}^{E}$ and $TS_{m}^{E}$ .

In the absence of the independent values of the activity coefficients, we cannot use the Gibbs–Duhem relation to test the thermodynamic consistency of the vapor pressure measurements. However, we can test the consistency of the $H_{m}^{E}$ and $G_{m}^{E}$ values via the Gibbs–Helmholtz equation. The $H_{m}^{E}$ values calculated at *T* = 298.15 K are shown as curves in Figure 7, together with our $H_{m}^{E}$ experimental data. The match can be considered satisfactory although considerable uncertainty is implied by the quantitative evaluation of $H_{m}^{E}$ from vapor pressures [72]. In the same figure and for the same temperature, $TS_{m}^{E}$ curves, obtained from $TS_{m}^{E}=H_{m}^{E}-G_{m}^{E}$ , are also plotted.

### 2.4. A Comparative Discussion of the Thermodynamic Excess Functions for Short-Chain Alcohol + 1,8-Cineol, or +di-n-Propylether

For comparative purposes, and in order to highlight the particularities of our binary mixture, the thermodynamic excess molar functions at *T* = 298.15 K and *x* = 0.5 of the short-chain alcohol + 1,8-cineole (cyclic ether) or +di-n-propylether (lineal ether) or +n-hexane (an inert solvent) are summarized in Table 7.

Among the reported values in Table 7, the low values of $H_{m}^{E}$ and $V_{m}^{E}$ for alcohol + 1,8-cineol mixtures stand out when comparing with those corresponding to the mixture of alcohol with di-n-propylether or with n-hexane. Such behavior can be qualitatively interpreted in terms of the type of interactions between the molecules that constitute the mixture, and the molecular shapes. Unlike what happens in the (alcohol + n-hexane) mixtures, where the most important contribution to the excess molar enthalpy is the breaking of hydrogen bonds in alcohol (endothermic contribution), in alcohol mixtures with both ethers we would also consider the breaking interactions of polar type in ether (endothermic contribution) and also the formation of alcohol—ether interactions (exothermic contribution). In the case of mixtures of alcohol with 1,8-cineole, we have to take into account that this molecule has a larger molar volume and a larger dipole moment than the corresponding di-n-propylether. For that reason, one would expect greater endothermic contributions to the excess molar enthalpy. However, experimental excess molar enthalpies are considerably lower in mixtures with 1,8-cineole than the corresponding ones with di-n-propylether. To justify this experimental behavior, we should consider that the alcohol-1,8-cineole (cyclic ether) interaction is stronger than the alcohol-di-n-propylether (linear ether) one. This justification is consistent with both the more negative values of the excess molar entropy and the excess molar volume of alcohol + 1,8-cineole mixtures, as shown in Table 7, as well as from the relative values of *λ*_ij_ interaction energies between 2-propanol-1,8-cineole and 2-propanol-di-n-propylether obtained from the vapor pressure data using Wilson’s model, as indicated above.

**Figure 8:**
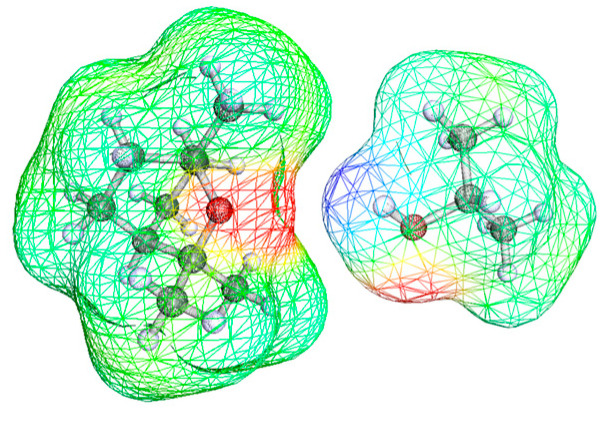
Favorable coupling between 1,8-cineole and 2-propanol molecules for the formation of a complex. The electronic charge density and the electrostatic potential surface were obtained using the software BIOVIA COSMOtherm 2020; Version 20.0.0 (Revision 5273M).

The negative value of $V_{m}^{E}$ and the low value of $H_{m}^{E}$ for alcohol–cineol mixtures probably also has to do with the slightly curled shape of the ether molecule, which can favor the formation of alcohol–ether complexes, thanks to a good spatial coupling of the molecules. A sketch of the possible coupling between molecules of 1,8-cineole and 2-propanol is represented in Figure 8. The figure shows the maps of the electrostatic potential of both geometrically optimized molecules, obtained via the software BIOVIA COSMOtherm 2020; Version 20.0.0 (Revision 5273M). For a mutual orientation as that represented in the figure, both the interaction between the oxygen atom -O- of 1,8-cineole with the highly positive H- atom of the hydroxyl -OH group of alcohol, and the interaction between the highly negative -O- oxygen atom of the hydroxyl –OH group of alcohol with the -H atoms of –CH_3_ groups of 1,8-cineole, would be favored.

The greater strength of the alcohol–cyclic ether interaction with respect to that existing in the case of the linear ether could be also caused by an increase in electron density of the oxygen atom in the cyclic molecule (ring strain), as pointed out by other authors [82,83]. To confirm the validity of this hypothesis, we calculated the electron density around the oxygen atom in both the molecules, 1,8-cineole and di-n-propylether, obtaining the values of 5.12 and 4.98 electrons, respectively, which it is consistent with previous arguments. The calculations of electron density have been carried out at the B3LYP/6-31** level of theory [84,85,86] and the analysis of the electron-pairing was conducted using the Electron Localization Function (ELF) methodology as implemented in the Topmod program [87].

In the discussion above, we are assuming that the main contribution to the alcohol–ether interaction is the hydrogen bond formation between the lone pair of the oxygen atom in the ether and the hydrogen atom of the OH group in the alcohol. This has been established by numerous authors, among which we could cite a recent article by Patel et al. on binary systems 1,8-cineol + cresol [88].

Focusing on the comparison between 2-propanol and the other alcohols included in Table 8, we can see that mixtures including 2-propanol show greater positive value of $H_{m}^{E}$ and appreciably less negative value of $V_{m}^{E}$ than mixtures where 2-propanol is replaced by one of the other alcohols. Only excess molar entropy in mixture 1,8-cineole with 2-propanol are quite similar to that of mixture with 1-propanol. The extra increase in the excess molar functions has been attributed to cyclic multimers formation in the case of branched alcohols in low polarity solvents [89].

### 2.5. Equations of State (EoS)

Table 8 shows the properties of the pure compounds used in this work in order to describe both the phase equilibrium and the volumetric behavior of 1-propanol (1) + 1,8-cineole (2) mixtures via the PRM, PRSV and SAFT models.

Equations for calculation are described in detail in Appendix A1 (EoS Implemented in PE) of the work of Pfohl, Petkov and Brunner [90].

The cubic PRM and PRSV EoS parameters for 2-propanol were calculated from the correlation of vapor pressure and saturation properties. The SAFT parameters for 2-propanol were taken from the literature [39]. The cubic PRM and PRSV EoS parameters, and SAFT parameters corresponding to 1,8-cineole were calculated in a previous paper [35].

**Table 8:** Pure component properties and parameters used for the application of the studied equations of state.

	*M*_w_/g·mol^−1^		*T*_b_/K	*T*_c_/K	*P*_c_/MPa	*ω*
1,8-cineole ^a^	154.25		449.6	661.12	3.019	0.338
2-propanol ^b^	60.096		355.4	508.3	4.760	0.665
**PRM-VT**	***P*_1_**		**c/b**		**Range *T*/K**	
1,8-cineole ^c^	−0.003518		−0.086491		278–450	
2-propanol ^d^	−0.256223		0.039731		278–493	
**PRSV-VT**	***k*_ap_**		**c/b**		**Range *T*/K**	
1,8-cineole ^c^	0.007355		−0.086427		278–450	
2-propanol ^d^	0.166735		0.039468		278–493	
**SAFT**	***m***	***υ*** **°°/L·mol^−1^**	***u*** **°·*k*^−1^/K**	***κ***	***ε*** **·*k*^−1^/K**	**Range *T*/K**
1,8-cineole ^c^	4.842	0.0178	263.43	-	-	278–450
2-propanol ^e^	3.249	0.0120	202.94	0.0210	2670	293–493

^a^ Ref. [32]. ^b^ Ref. [91]. ^c^ Ref. [35]. ^d^ This work. ^e^ Ref. [39].

The van der Waals one-fluid mixing rules [43] were used to determine the *PρT* behavior of the mixtures. Classical quadratic combining rules for the cross-terms [43] were selected in all cases. A quadratic dependence between the interaction parameter, *k*_ij_, and the temperature was found in the experimental range considered.

The *k*_ij_ interaction parameter has been set to our VLE data, showing a quadratic dependence on temperature. The fitted parameters for the equation,

*k*_ij_ = *a* + *b*·*T*/K + *c*·*T^2^*/K^2^ (14)

appear in Table 9 together with the regression coefficients.

**Figure 9:**
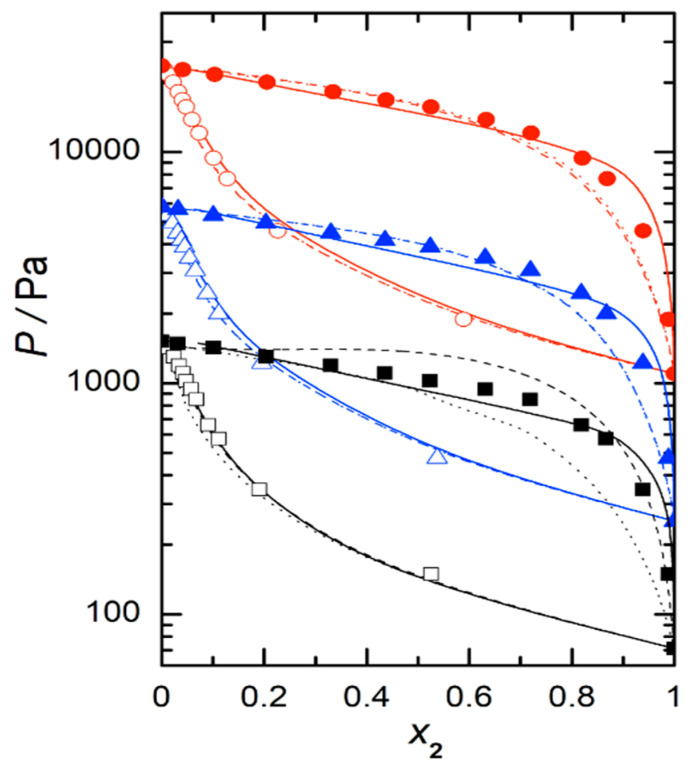
Isothermal vapor liquid equilibrium of the 2-propanol (1) + 1,8-cineole (2) system. Full symbols experimental data: (■), *T* = 278.15 K; (▲), *T* = 298.15 K; (●), and *T* = 323.15 K. Open symbols were obtained from the Wilson equation. Lines, EOS correlations: – – –, PRM-VT; ······, PRSV-VT; ―, SAFT.

Figure 9 shows the experimental VLE at three temperatures together with the obtained results using the selected EoS. It should be noted that bubble curves corresponding to the three models appear well separated for the temperatures of 278.15 and 323.15 K, but not at 298.15 K, where the bubble curves for PRM and PRSV models appear to be almost overlapping. A similar behavior is displayed in the dew curves for these two same models at 298.15 K. The dew curves corresponding to the PRM and SAFT models at 278.15 K and those of the PRM and PRSV ones at 323.15 K are also practically coincident. The best results for the correlations of the experimental data of the mixture under study were achieved with PRSV-VT and PRM-VT. The absolute average percentage deviation values (ADD) for these models were 9.27% and 10.99%, respectively. The ADD obtained for SAFT was 19.30%.

**Figure 10:**
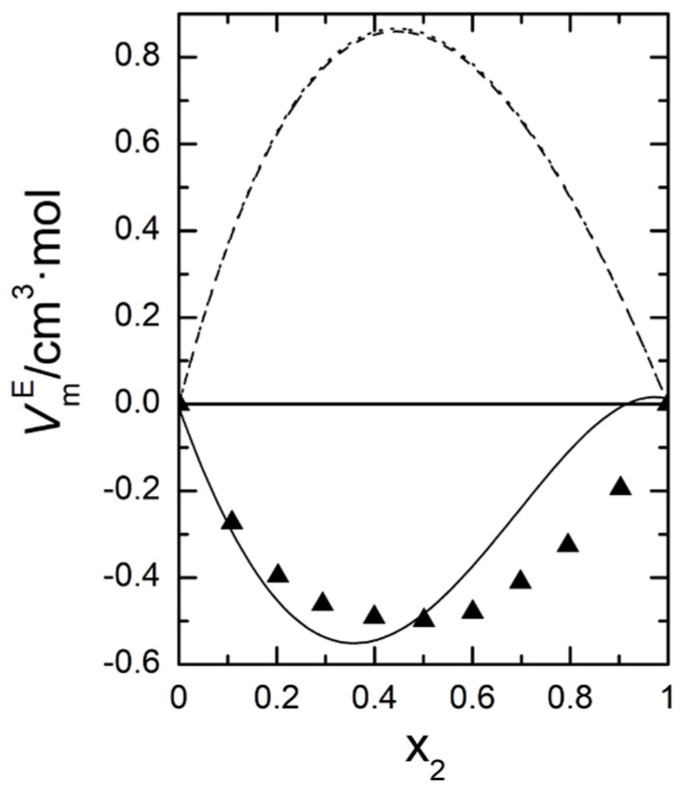
Volumetric behavior of the 2-propanol (1) + 1,8-cineole (2) system at 298.15 K. (▲), This work; – – –, PRM-VT; ········, PRSV-VT; ―, SAFT.

The major or minor capacity of the three EoS to reproduce the volumetric behavior of the system was also tested at 298.15 K. Figure 10 shows our experimental data for the excess molar volume at that temperature, together with the predictions of the three EoS tested.

As it can be observed, only the SAFT model correctly reproduces the sign of $V_{m}^{E}$ , and even approaches the values of the volumetric behavior of the system quite well, something that the other two EoS cannot satisfy even when the refinement of the volume translation is used in them.

## 3. Materials and Methods

### 3.1. Chemicals

2-Propanol and 1,8-cineole were supplied by Aldrich, Seelze, Germany (mass fraction purity > 0.999 and >0.990, respectively). All the chemicals were low water content, stored over molecular sieve (4 Å), and used without further purification. The mass fraction purity was checked via gas chromatography and found to be 0.9999 for 2-propanol, and 0.9970 for 1,8-cineole.

### 3.2. Apparatus and Procedures

Vapor pressure measurements were carried out according a static method, using an apparatus similar to that of Marsh [92], but with the incorporation of some different details. The device and operating method have been thoroughly described previously [93]. Several important points should be noted here. The undesirable effects of condensation on the mercury meniscus were avoided by circulating the thermostat water at ±0.1 K to maintain the manometer temperature at 325 K. In the same way, most vapor phase was maintained at that temperature, *T* = 325 K. Liquid sample temperature was measured using Beckmann thermometers, calibrated against vapor-liquid equilibria (VLE) of pure benzene (Merck mole fraction > 0.999 and distilled twice), along with Ambrose’s equation [94] relating temperature with pressure by means of a sum of Chebyshev polynomials up to the sixth degree. Thus, the accuracy in the temperature measurements was estimated to be ±0.01 K. The volume of the cell containing the liquid mixture was about 12 cm^3^, and (8 to 10) cm^3^ of liquid were used in each experiment. Previously, to be successively added by gravity into the cell immersed in liquid nitrogen, the liquids were degassed via magnetic stirring, with the (air plus vapor) phase being pumped away periodically. Masses of both components were determined by weighing with a precision electronic balance (0.0001 g). Caution was taken to prevent evaporation. Conversion to molar quantities was based on the relative atomic mass table issued by IUPAC, leading to an uncertainty in the mole fraction estimated to be less than ±0.0003. The vapor pressures, P, obtained from the difference in heights of the mercury meniscus in the two columns of the manometer, were calculated using the specific weight of that element for the value of gravitational acceleration at the laboratory where the measurements were performed. Manometer readings were performed using a Wild KM-305 cathetometer within ±0.01 mm, and pressure reproducibility was estimated to be better than 13 Pa. The uncertainty in the vapor pressure is estimated to be less than 0.1%.

Advantages and limitations of the static (isothermal) measurements method used in this work have been previously detailed by Smith and Menzies [95], together with the more general sources of error involved in steam pressure measurement, and particular reference to the individual sources of error in both the static and dynamic methods. Despite their greater laboriousness, compared to dynamic measurements, the main advantages of the static ones are not having to resort to physical or chemical analysis of the phases, directly providing the value of the Gibbs energy at each of the measured temperatures and avoiding inaccuracies derived from the accumulation of impurities throughout the measurement process. Regarding the more general sources of error involved in any of the measurement methods, both static and dynamic, those authors have highlighted deficiencies in (i) the stability, distribution and precise determination of temperature, (ii) the measurement and corrections for pressure measurements, and (iii) the presence of impurities in the chemical species used as components. In our work, we tried to minimize these deficiencies through methodological details such as those indicated above. Finally, and from the point of view of thermodynamic consistence compliance based on the Gibbs–Duhem equation, the static (isothermal) measurements are also more adequate than the dynamic (isobaric) ones.

A vibrating tube densimeter DMA 5000, Anton Paar GmbH, Graz, Austria was used for density measurements on the pure liquids and mixtures. The sample density is calculated from the vibration period with an uncertainty of ±0.04 kg·m^−3^. The composition of the binary mixtures was determined by weighing the vapor pressure mixtures preparation in a similar way. From the experimental densities, the excess molar volumes were calculated. The uncertainty in excess molar volumes is estimated to be ±2·× 10^−9^ m^3^·mol^−1^.

A Thermometric 2277 Thermal Activity Monitor, American Laboratory Trading, San Diego, (CA, USA) together with two Shimadzu (model LC-10ADVP HPLC), Shimadzu Europe GmbH, Duisburg, Germany variable speed piston pumps, was used to determine excess enthalpies at the different temperatures. The pumps were programmed in order to be able to measure the excess molar enthalpies at the selected molar fractions of mixture and they were previously calibrated. The uncertainty in mole fraction is estimated to be ±0.001, and the uncertainty in $H_{m}^{E}$ measurements is better than 2%, as verified by comparing the results for a standard system with those of the reference system [96]. Some additional details can be found in a previous paper [97].

In Table 10, experimental and literature densities, *ρ*, of pure liquids at the different temperatures are showed. The agreement between both sets of values could be considered as satisfactory.

## 4. Conclusions

New isothermal vapor–liquid equilibrium data at three temperatures, from 278.15 K to 323.15 K, and over the entire composition range for the binary mixture 2-propanol + 1,8-cineole are presented. Vapor pressures have been measured via a static method. From these data, activity coefficients and excess Gibbs energies have been calculated using Barker’s method and the Wilson equation. The consistency of the obtained vapor pressure data is substantiated by the close values of excess molar enthalpies calculated from the vapor pressures by applying the Gibbs–Helmholtz equation to those experimental values obtained in the present work. Although the system is far from an ideal behavior, showing a large positive deviation from Raoult’s law, azeotrope do not appear within the considered temperature interval.

On the other hand, excess molar volumes for this system at temperatures between 288.15 and 318.15 K have been calculated from the densities that were experimentally obtained.

A brief comparison is presented between the thermodynamic behavior of short-chain alcohol + 1,8-cineol binary mixtures and those where the cyclic ether has been replaced by a linear ether, di-n-propylether or inert solvent, n hexane. This comparison allowed us to attribute a higher OH–O interaction energy in the alcohol + cyclic ether mixture than that corresponding to the mixture with linear ether. This effect is probably due to an increase in the electronic density of the oxygen atom in the cyclic ether (which is associated with the ring strain) together with a steric effect derived from the possibility of a closer coupling between the alcohol molecule and the cyclic ether molecule.

Both experimental vapor-liquid equilibrium (VLE) and volumetric data were correlated using three different thermodynamic models, namely PRSV-VT, PRM-VT and SAFT. The best results for the correlations of the experimental VLE data are achieved when the first two models are used. This is probably due to the regression flexibility conferred in the cases of the modified PR models by considering the binary interaction parameter *k*_ij_ as an adjustable coefficient. On the contrary, only the SAFT model approaches the volumetric behavior of the real system better than the other EoS, even using the volume translation correction in them. The detail of the localized interactions between the areas of higher electron charge densities that SAFT considers could be responsible for that improvement. The approximations reported, as provided by the two considered versions of the Robinson–Stokes equation for VLE, and the one provided by SAFT for excess enthalpy, are within a very reasonable range.

Because of the special characteristics of the chemicals involved in this study, the results obtained in this work can be a good database in the development of advanced theoretical models such as molecular dynamics and direct simulation Monte Carlo. From an application point of view, these results, together with others of a more industrial type, can contribute to the development of biorefineries. More concretely, the values of parameters, as well as the reported experimental data, can be potentially suitable for simulation and for designing separation processes to obtain the interesting chemical 1,8-cineole.

## Figures and Tables

**Table 1:** Molar volumes, *V*^0^, and vapor pressures, *P*^0^, of pure liquids used in the Barker analysis ^a^, and data taken from the literature.

	2-Propanol			1,8-Cineole		
	*V*^0^ × 10^6^/m^3^·mol^−1^	*P*^0^/Pa		*V*^0^ × 10^6^/m^3^·mol^−1^	*P*^0^/Pa	
*T*/K	lit. ^b,c^	exp. ^b^	lit. ^d^	lit. ^b,e^	exp. ^b^	lit.
278.15	75.35	1521	1514	164.6	71	54.80 ^g^
283.15	75.70	2164	2162	165.3	93	80.12 ^g^
283.51	-	-	-	-	-	83.55 ^h^
288.15	76.12	3069	3044	166.1	130	133 ^f^/115.17 ^g^
293.15	76.52	4212	4228	166.9	178	163.72 ^g^
293.49	-	-	-	-	-	170.01 ^h^
298.15	76.93	5781	5797	167.7	253	229.50 ^g^
303.15	77.35	7851	7852	168.5	346	317.05 ^g^
303.34	-	-	-	-	-	323.77 ^h^
308.15	77.78	10,515	10,511	169.2	477	431.34 ^g^
313.15	78.23	13,934	13,913	170.0	627	-
313.23	-	-	-	-	-	591.71 ^h^
318.15	78.66	18,242	18,220	170.9	828	-
323.15	79.15	23,623	23,618	171.7	1101	-

^a^ Standard uncertainty u is u(*T*) = 0.01 K and the combined expanded uncertainty *U*_c_ is *U*_c_(*P*) = 0.1% with a 0.95 level of confidence (*k* = 2). ^b^ Used in the Barker analysis. ^c^ Ref. [64]. ^d^ Ref. [65]. ^e^ Ref. [35]. ^f^ Ref. [66]. ^g^ Ref. [31]. ^h^ Ref. [67].

**Table 2:** Values of the vapor pressure *P*, deviations Δ*P* = *P* − *P_calc_*, activity coefficients, *γ*_1_ and *γ*_2_, and excess molar Gibbs energies, $G_{m}^{E}$ ^a^, for the mixtures 2-propanol (1) + 1,8-cineole (2).

*x*_2_	*P*/Pa	∆*P*/Pa	*γ*_1_	*γ*_2_	$G_{m}^{E}/J\cdot {\mathrm{mol}}^{-1}$	*x*_2_	*P*/Pa	∆*P*/Pa	*γ*_1_	*γ*_2_	$G_{m}^{E}/J\cdot {\mathrm{mol}}^{-1}$
2-Propanol (1) + 1,8-Cineole (2)											
*T*/K *=* 278.15											
0.0309	1485	3	1.0012	2.9938	81	0.6307	944	6	1.5749	1.1958	649
0.1004	1425	22	1.0120	2.5666	244	0.7188	849	13	1.8228	1.1146	571
0.2031	1303	1	1.0488	2.1015	437	0.8182	661	−16	2.2277	1.0494	428
0.3294	1195	1	1.1308	1.7073	598	0.8670	576	7	2.4999	1.0271	335
0.4351	1107	−2	1.2377	1.4756	670	0.9382	347	−5	3.0297	1.0061	172
0.5233	1024	−13	1.3622	1.3297	686	0.9882	149	16	3.5396	1.0002	35
*T*/K *=* 283.15											
0.0310	2128	20	1.0012	2.9499	82	0.6308	1323	2	1.5661	1.1918	650
0.1006	2007	14	1.0119	2.5340	245	0.7189	1176	2	1.8084	1.1121	572
0.2032	1865	19	1.0482	2.0801	439	0.8182	940	−5	2.2020	1.0483	429
0.3295	1684	−6	1.1293	1.6938	601	0.8670	808	17	2.4652	1.0264	336
0.4352	1569	1	1.2347	1.4666	673	0.9382	471	−14	2.9741	1.0060	172
0.5234	1451	−13	1.3573	1.3233	688	0.9882	183	3	3.4600	1.0002	35
*T*/K *=* 288.15											
0.0313	3010	22	1.0012	2.9026	82	0.6309	1859	−1	1.5565	1.1875	651
0.1006	2838	13	1.0117	2.4997	246	0.7190	1655	6	1.7930	1.1095	572
0.2032	2622	7	1.0476	2.0570	440	0.8183	1329	6	2.1748	1.0471	428
0.3297	2398	7	1.1277	1.6793	602	0.8671	1112	7	2.4285	1.0257	335
0.4353	2205	−11	1.2314	1.4569	674	0.9382	657	−17	2.9160	1.0058	171
0.5235	2056	−10	1.3519	1.3165	689	0.9882	253	3	3.3775	1.0002	35
*T*/K *=* 293.15											
0.0316	4112	12	1.0012	2.8844	84	0.6310	2564	22	1.5514	1.1811	651
0.1007	3892	15	1.0119	2.4806	249	0.7191	2274	28	1.7811	1.1052	570
0.2033	3574	−15	1.0480	2.0388	444	0.8184	1800	8	2.1471	1.0450	426
0.3299	3264	−17	1.1283	1.6638	607	0.8672	1469	−22	2.3875	1.0245	333
0.4354	3010	−28	1.2315	1.4448	677	0.9383	896	−10	2.8430	1.0055	170
0.5236	2828	−2	1.3506	1.3070	691	0.9882	321	−16	3.2674	1.0002	34
*T*/K *=* 298.15											
0.0321	5654	29	1.0012	2.8630	87	0.6312	3492	12	1.5461	1.1752	651
0.1009	5320	−2	1.0121	2.4607	252	0.7193	3078	11	1.7695	1.1014	569
0.2035	4936	6	1.0484	2.0208	448	0.8185	2446	9	2.1213	1.0431	424
0.3302	4481	−25	1.1288	1.6490	611	0.8673	2003	−21	2.3501	1.0234	331
0.4356	4154	−18	1.2312	1.4336	680	0.9384	1221	−5	2.7782	1.0052	168
0.5238	3894	12	1.3491	1.2983	692	0.9882	475	10	3.1715	1.0002	34
*T*/K *=* 303.15											
0.0329	7621	−14	1.0013	2.8303	89	0.6314	4713	9	1.5397	1.1707	652
0.1013	7215	−10	1.0121	2.4362	255	0.7195	4153	16	1.7580	1.0986	569
0.2038	6699	8	1.0484	2.0025	451	0.8187	3313	38	2.0988	1.0418	423
0.3307	6105	−7	1.1285	1.6356	614	0.8675	2676	−37	2.3189	1.0226	330
0.4358	5640	−17	1.2298	1.4243	683	0.9384	1623	−17	2.7274	1.0050	168
0.5241	5248	−10	1.3462	1.2914	694	0.9883	633	7	3.0988	1.0002	34
*T*/K *=* 308.15											
0.0325	10,218	−11	1.0013	2.8076	89	0.6318	6249	−27	1.5338	1.1660	652
0.1017	9658	−16	1.0122	2.4119	257	0.7199	5537	30	1.7469	1.0956	569
0.2041	8951	−10	1.0485	1.9839	455	0.8189	4353	7	2.0764	1.0404	422
0.3315	8170	−11	1.1286	1.6216	618	0.8677	3589	−4	2.2877	1.0218	329
0.4362	7557	−11	1.2288	1.4145	685	0.9386	2140	−30	2.6763	1.0048	167
0.5245	7054	27	1.3437	1.2841	696	0.9883	871	28	3.0260	1.0002	34
*T*/K *=* 313.15											
0.0338	13,490	−48	1.0014	2.7686	93	0.6324	8269	10	1.5272	1.1610	651
0.1015	12,790	−27	1.0122	2.3878	258	0.7204	7246	21	1.7346	1.0924	567
0.2039	11,878	11	1.0481	1.9658	456	0.8193	5721	44	2.0524	1.0389	420
0.3310	10,824	−2	1.1274	1.6093	619	0.8680	4656	−24	2.2547	1.0210	326
0.4368	9977	−20	1.2274	1.4039	686	0.9387	2780	−36	2.6232	1.0046	165
0.5251	9254	−16	1.3408	1.2763	696	0.9883	1072	−26	2.9512	1.0002	33
*T*/K *=* 318.15											
0.0363	17,673	−13	1.0016	2.7299	100	0.6319	10780	15	1.5195	1.1565	650
0.1024	16,765	−2	1.0124	2.3622	262	0.7199	9481	84	1.7204	1.0896	565
0.2045	15,507	−19	1.0485	1.9459	460	0.8198	7322	−12	2.0291	1.0373	417
0.3322	14,127	−25	1.1281	1.5933	622	0.8684	5993	−37	2.2218	1.0200	323
0.4365	13,040	−31	1.2259	1.3944	687	0.9389	3592	−26	2.5692	1.0044	163
0.5247	12,103	−6	1.3373	1.2693	696	0.9883	1432	7	2.8747	1.0002	33
*T*/K *=* 323.15											
0.0410	22,785	−29	1.0020	2.6720	113	0.6328	13851	−11	1.5135	1.1516	649
0.1037	21,678	−11	1.0127	2.3316	266	0.7206	12092	22	1.7092	1.0867	563
0.2055	20,080	−7	1.0487	1.9245	463	0.8204	9415	32	2.0078	1.0358	414
0.3341	18,256	−32	1.1287	1.5768	625	0.8689	7677	−23	2.1926	1.0192	321
0.4375	16,849	−38	1.2249	1.3840	689	0.9392	4569	−50	2.5224	1.0042	162
0.5257	15,679	54	1.3349	1.2616	696	0.9883	1889	38	2.8096	1.0002	33

^a^ Standard uncertainties, u, are u(*x*) = 0.0001, u(*T*) = 0.01 K, and the combined expanded uncertainty, *U*_c_, is *U*_c_(*P*) = 0.1% with a 0.95 level of confidence (*k* = 2).

**Table 3:** Wilson parameters, *Λ*_12_ and *Λ*_21_, standard deviations, *s*(Pa), and Wilson coefficients, *λ*_12_ − *λ*_11,_ (J·mol^−1^) and *λ*_12_ − *λ*_22_ (J·mol^−1^) of Equations (2) and (4)–(7).

	2-Propanol (1) + 1,8-Cineole (2)				
*T*/K	*Λ*_12_	*Λ*_21_	*s*/Pa	*λ*_12_ − *λ*_11_	*λ*_12_ − *λ*_22_
278.15	0.4255	0.5511	12	3783 (3815)	−429 (−460)
283.15	0.4341	0.5548	12	3803 (3800)	−452 (−452)
288.15	0.4434	0.5589	11	3818 (3784)	−476 (−443)
293.15	0.4633	0.5504	19	3776 (3768)	−445 (−434)
298.15	0.4815	0.5434	16	3743 (3752)	−420 (−425)
303.15	0.4942	0.5417	20	3739 (3737)	−417 (−416)
308.15	0.5082	0.5390	21	3725 (3721)	−408 (−407)
313.15	0.5227	0.5371	28	3710 (3705)	−402 (−399)
318.15	0.5401	0.5320	33	3682 (3689)	−383 (−390)
323.15	0.5543	0.5301	34	3666 (3674)	−375 (−381)

**Table 4:** Excess molar enthalpies, $H_{m}^{E}$ , for the 2-propanol + 1,8-cineole system at four temperatures ^a^.

*x*_2_	$H_{m}^{E}/Jmol^{-1}$			
	288.15 K	298.15 K	308.15 K	318.15 K
0.000	0	0	0	0
0.050	65	71	77	88
0.102	146	160	175	196
0.206	291	325	360	401
0.303	396	448	495	554
0.409	474	542	609	676
0.510	517	599	663	749
0.606	518	602	683	762
0.703	483	558	644	719
0.804	401	461	530	593
0.897	245	283	334	368
0.952	124	143	172	195
1.0000	0	0	0	0

^a^ Standard uncertainties, u, are u(*x*_2_) = 0.001, u(*T*) = 0.01 K and the combined expanded uncertainty, *U*_c,_ is *U*_c_( $H_{m}^{E}$ ) = 2% with a 0.95 level of confidence (*k* = 2).

**Table 5:** Densities for the 2-propanol + 1,8-cineole system at four temperatures ^a^.

*x*_2_	*ρ*/kg·m^−3^			
	288.15 K	298.15 K	308.15 K	318.15 K
0.0000	789.34	780.99	772.37	763.40
0.1084	821.21	812.72	803.98	794.93
0.2025	842.65	834.07	825.27	816.20
0.2938	859.68	851.02	842.19	833.12
0.3996	875.90	867.22	858.36	849.56
0.5011	888.92	880.21	871.35	862.32
0.6004	899.70	890.97	882.13	873.15
0.6980	908.60	899.88	891.06	882.14
0.7953	916.30	907.60	898.81	889.96
0.9029	923.59	914.91	906.19	897.44
1.0000	928.96	920.34	911.73	903.13

^a^ Standard uncertainties, u, are u(*x*_2_) = 0.0001, u(*T*) = 0.01 K and the combined expanded uncertainty, *U*_c,_ is *U*_c_(*ρ*) = 0.04 kg·m^−3^ with a 0.95 level of confidence (*k* = 2).

**Table 6:** Coefficients and standard deviations, $s\left(Q_{m}^{E}\right)$ , for least squares representation by Equation (2) of $\;H_{m}^{E}$ (J·mol^−1^) and $V_{m}^{E}$ (cm^3^·mol^−1^) at the four temperatures studied.

	$Q_{m}^{E}$	*A*_0_	*A*_1_	*A*_2_	*A*_2_	$s(Q_{m}^{E})$
288.15 K	$H_{m}^{E}$	2063	−617	136		6.3
	$V_{m}^{E}$	−2.017	−0.227	−0.770	−0.25	0.007
298.15 K	$H_{m}^{E}$	2380	−768	75		6.6
	$V_{m}^{E}$	−1.979	−0.247	−0.765	−0.300	0.006
308.15 K	$H_{m}^{E}$	2667	−981	197		7.8
	$V_{m}^{E}$	−1.912	−0.306	−0.737	−0.31	0.006
318.15 K	$H_{m}^{E}$	2978	−1107	204		7.0
	$V_{m}^{E}$	−1.847	−0.378	−0.642	−0.30	0.007

**Table 7:** Thermodynamic excess functions for {0.5 alkanol + 0.5 1,8-cineole or +0.5 di-n-propylether or +n-hexane} at 298.15 K.

Alkanol	1,8-Cineole ^a^ (*μ* = 1.544 D) ^b^			Di-n-Propylether (*μ* = 1.2 D) ^d^			n-Hexane ^h^		
	$H_{m}^{E}$	$TS_{m}^{E}$	$V_{m}^{E}$	$H_{m}^{E}$	$TS_{m}^{E}$	$V_{m}^{E}$	$H_{m}^{E}$	$TS_{m}^{E}$	$V_{m}^{E}$
	J·mol^−1^		cm^3^·mol^−1^	J·mol^−1^		cm^3^·mol^−1^	J·mol^−1^		cm^3^·mol^−1^
Ethanol	229	−501	−0.645	717 ^e^	−254 ^f^	−0.294 ^e^	555	−850	0.410
1-Propanol	160	−384	−0.700	741 ^g^	−80 ^g^	−0.388 ^g^	565	−680	0.180
2-Propanol	600 ^c^	−400 ^c^	−0.495 ^c^	956 ^g^	102 ^g^	−0.027 ^g^	787 ^i^	−270 ^j^	0.510 ^k^
1-Butanol	213	−254	−0.664	743 ^e^	4 ^f^	−0.468 ^e^	510	−630	0.080

^a^ Ref. [33]. ^b^ Refs. [73,74]. ^c^ This work. ^d^ Refs. [75,76]. ^e^ Ref. [77]. ^f^ Ref. [63]. ^g^ Ref. [62]. ^h^ Ref. [78]. ^i^ Ref. [79]. ^j^ G^E^—55 value calculated at 303.15 *k* from vapor pressures data taken from Barraza and Edwards [80]. ^k^ Ref. [81].

**Table 9:** Values of the coefficients *a*, *b* and *c* in Equation (14) and regression coefficient, *R*^2^.

Model	*a*	*b*	*c*	*R* ^2^
PRM-VT	3.4140	−2.1981 × 10^−2^	3.5557 × 10^−5^	0.954
PRSV-VT	1.3550	−8.9000 × 10^−3^	1.4854 × 10^−5^	0.964
SAFT	0.5930	−4.0773 × 10^−3^	6.9106 × 10^−6^	0.976

**Table 10:** Experimental and literature densities of pure liquids ^a^.

	*ρ*/kg·m^−3^			
	2-Propanol		1,8-Cineole	
T/K	Experimental	Literature	Experimental	Literature
288.15	789.34	-	928.96	928.78 ^g^
298.15	780.99	780.98 ^b^ 780.82 ^c^ 781.26 ^d^	920.34	920.29 ^g^
308.15	772.37	772.51 ^e^ 772.6 ^f^	911.73	-
318.15	763.40	-	903.13	-

^a^ Standard uncertainty, u, is u(*T*) = 0.01 K and the combined expanded uncertainty, *U*_c_, is *U*_c_(*ρ*) = 0.04 kg·m^−3^ with a 0.95 level of confidence (*k* = 2). ^b^ Ref. [98]. ^c^ Ref. [99]. ^d^ Ref. [64]. ^e^ Ref. [100]. ^f^ Ref. [101].^g^ Ref. [102].

## Data Availability

All data are contained within the article.

## References

[1] Cai Z.M., Peng J.Q., Chen Y., Tao L., Zhang Y.Y., Fu L.Y., Long Q.D., Shen X.C. (2020). 1,8-Cineole: A review of source, biological activities, and application. J. Asian Nat. Prod. Res..

[2] De Vincenzi M., Mancini E., Dessi M.R. (1996). Monographs on botanical flavouring substances used in foods. Part V. Fitoterapia.

[3] Scientific Committee on Food, European Commission Health and Consumer Protection Directorate-General (2002). Opinion of the Scientific Committee on Food on Eucalyptol.

[4] Bhowal M., Gopal M. (2015). Eucalyptol: Safety and Pharmacological Profile. RGUHS J. Pharm. Sci..

[5] Majed M.M., Shadi F.G., Karem H.A., Al-Azzam S.I., Wasfi M.O. (2013). Antimicrobial Activity of Common Mouthwash Solutions on Multidrug-Resistance Bacterial Biofilms. J. Clin. Med. Res..

[6] Worth H., Schacher C., Dethlefsen U. (2009). Concomitant therapy with Cineole (*Eucalyptole*) reduces exacerbations in COPD: A placebo-controlled double-blind trial. Respir. Res..

[7] Juergens U.R. (2014). Anti-inflammatory properties of the monoterpene 1.8-cineole: Current evidence for co-medication in inflammatory airway diseases. Drug Res..

[8] Van Vuuren S.F., Viljoen A.M. (2007). Antimicrobial activity of limonene enantiomers and 1,8-cineole alone and in combination. Flavour Fragr. J..

[9] Merghni A., Noumi E., Hadded O., Dridi N., Panwar H., Ceylan O., Mastouri M., Snoussi M. (2018). Assessment of the antibiofilm and antiquorum sensing activities of Eucalyptus globulus essential oil and its main component 1,8-cineole against methicillin-resistant Staphylococcus aureus strains. Microb. Pathog..

[10] Morcia C., Malnati M., Terzi V. (2012). In vitro antifungal activity of terpinen-4-ol, eugenol, carvone, 1,8-cineole (eucalyptol) and thymol against mycotoxigenic plant pathogens. Food Addit. Contam. Part A-Chem..

[11] Kin K.Y., Seo H.J., Min S.S., Park M., Seol G.H. (2014). The effect of 1,8-cineole inhalation on preoperative anxiety: A randomized clinical trial. Evid. Based Complement. Altern. Med..

[12] Heard C.M., Kung D., Thomas C.P. (2006). Skin penetration enhancement of mefenamic acid by ethanol and 1,8-cineole can be explained by the ‘pull’ effect. Int. J. Pharm..

[13] Dougnon G., Ito M. (2020). Inhalation Administration of the Bicyclic Ethers 1,8- and 1,4-cineole Prevent Anxiety and Depressive-Like Behaviours in Mice. Molecules.

[14] Abdalla A.N., Shaheen U., Abdallah Q.M.A., Flamini G., Bkhaitan M.M., Abdelhady M.I.S., Ascrizzi R., Bader A. (2020). Proapoptotic Activity of Achillea membranacea Essential Oil and Its Major Constituent 1,8-Cineole against A2780 Ovarian Cancer Cells. Molecules.

[15] Rodenak-Kladniew B., Castro M.A., Crespo R., Galle M., de Bravo M.G. (2020). Anti-cancer mechanisms of linalool and 1,8-cineole in non-small cell lung cancer A549 cells. Heliyon.

[16] Paul K., Ganguly U., Chakrabarti S., Bhattacharjee P. (2020). 1,8-Cineole-Rich Extract of Small Cardamom Seeds More Effective in Preventing Alzheimer’s Disease than 1,8-Cineole Alone?. Neuromol. Med..

[17] An F., Bai Y., Xuan X., Bian M., Zhang G., Wei C. (2022). 8-Cineole Ameliorates Advanced Glycation End Products-Induced Alzheimer’s Disease-like Pathology In Vitro and In Vivo. Molecules.

[18] Sharma A.D., Kaur I. (2022). Targeting UDP-Glycosyltransferase, Glucosamine-6-Phosphate Synthase and Chitin Synthase by Using Bioactive 1,8 Cineole for “Aspergillosis” Fungal Disease Mutilating COVID-19 Patients: Insights from Molecular Docking, Pharmacokinetics and In-vitro Studies. Chem. Afr..

[19] Hamiche S., Bouzidi N., Daghbouche Y., Badis A., Garrigues S., de la Guardia M., El Hattab M. (2018). Eucalyptol-based green extraction of brown alga Zonaria tournefortii. Sustain. Chem. Pharm..

[20] Campos J.F., Scherrmann M.C., Berteina-Raboin S. (2019). Eucalyptol: A new solvent for the synthesis of heterocycles containing oxygen, sulfur and nitrogen. Green Chem..

[21] Eisenbrand G., Cohen S.M., Fukushima S., Gooderham N.J., Guengerich F.P., Hecht S.S., Taylor S.V. (2021). FEMA GRAS assessment of natural flavor complexes: Eucalyptus oil and other cyclic ether-containing flavoring ingredients. Food Chem. Toxicol..

[22] Sánchez-Vicente Y., Cabañas A., Renuncio J.A.R., Pando C. (2013). Supercritical CO_2_ as a green solvent for eucalyptus and citrus essential oils processing: Role of thermal effects upon mixing. RSC Adv..

[23] Prat D., Hayler J., Wells A. (2014). A survey of solvent selection guides. Green Chem..

[24] Samudrala S., Bhattacharya S. (2018). Toward the Sustainable Synthesis of Propanols from Renewable Glycerol over MoO_3_-Al_2_O_3_ Supported Palladium Catalysts. Catalysts.

[25] Wang K., Strobel G., Yan D.H. (2017). The Production of 1,8-Cineole, a Potential Biofuel, from an Endophytic Strain of *Annulohypoxylon* sp. FPYF3050 When Grown on Agricultural Residues. J. Sustain. Bioenergy Syst..

[26] Tasoren E., Aydogan H., Gokmen M.S. (2021). Research of effect on gasoline-2-propanol blends on exhaust emission of gasoline engine with direct injection using Taguchi approach. Eur. Mech. Sci..

[27] Weberdemenezes E., Dasilva R., Cataluna R., Ortega R. (2006). Effect of ethers and ether/ethanol additives on the physicochemical properties of diesel fuel and on engine tests. Fuel.

[28] Raveendran P., Zimmermann D., Häber T., Suhm M.A. (2000). Exploring a hydrogen-bond terminus: Spectroscopy of eucalyptol–alcohol clusters. Phys. Chem. Chem. Phys..

[29] Du L., Tang S., Hansen A.S., Frandsen B.N., Maroun Z., Kjaergaard H.G. (2017). Subtle differences in the hydrogen bonding of alcohol to divalent oxygen and sulfur. Chem. Phys. Lett..

[30] Aparicio S., Alcalde R., Dávila M.J., García B., Leal J.M. (2007). Properties of 1,8-Cineole: A Thermophysical and Theoretical Study. J. Phys. Chem. B.

[31] Štejfa V., Fulem M., Růžička K., Červinka C. (2014). Thermodynamic study of selected monoterpenes III. J. Chem. Thermodyn..

[32] Lasarte J.M., Martín L., Langa E., Urieta J.S., Mainar A.M. (2008). Setup and Validation of a P ρ T Measuring Device. Volumetric Behavior of the Mixture 1,8-Cineole + Ethanol. J. Chem. Eng. Data.

[33] Alfaro P., Langa E., Martínez-López J.F., Urieta J.S., Mainar A.M. (2010). Thermophysical properties of the binary mixtures (1,8-cineole + 1-alkanol) at T = (298.15 and 313.15) K and at atmospheric pressure. J. Chem. Thermodyn..

[34] Torcal M., García-Abarrio S., Pardo J.I., Mainar A.M., Urieta J.S. (2010). P, ρ, T Measurements and Isobaric Vapor-Liquid-Equilibria of the 1,3,3-Trimethyl-2-oxabicycle[2,2,2]octane + Propan-1-ol Mixture: Cubic and Statistical Associating Fluid Theory-Based Equation of State Analysis. J. Chem. Eng. Data.

[35] Gimeno B., Torcal M., Mainar A.M., Pérez P. (2011). Isothermal Vapor-Liquid Equilibrium of (1-butanol + 1,8-cineole) at Ten Temperatures between 278.15 K and 323.15 K. J. Chem. Eng. Data.

[36] Gimeno B., Torcal M., Mainar A.M., Urieta J.S., Pérez P. (2011). Total vapour pressure and excess Gibbs energy of ethanol with 1,8-cineole at temperatures between 278.15 K and 323.15 K. Fluid Phase Equilib..

[37] Gimeno B., Martínez S., Urieta J.S., Pérez P. (2012). Vapor Pressures and Activity Coefficients of (1-propanol + 1,8-cineole) at 10 Temperatures between 278.15 K and 323.15 K. J. Chem. Eng. Data.

[38] Peng D., Robinson D.B. (1976). A New Two-Constant Equation of State. Ind. Eng. Chem. Fundam..

[39] Huang S.H., Radosz M. (1990). Equation of State for Small, Large, Polydisperse and Associating Molecules. Ind. Eng. Chem. Res..

[40] van der Waals J.D. (1873). Over de Continuiteit van den Gas en Vloeistoftoestand. Ph.D. Thesis.

[41] Papadopoulos A.I., Tsivintzelis I., Seferlis P., Linke P., Reedijk J. (2018). Computer-aided molecular design: Fundamentals, methods, and applications. Elsevier Reference Module in Chemistry, Molecular Sciences and Chemical Engineering.

[42] Lopez-Echeverry J.S., Reif-Acherman S., Araujo-Lopez E. (2017). Peng-Robinson equation of state: 40 years through cubics. Fluid Phase Equilib..

[43] Valderrama J.O. (2003). The state of the cubic equations of state. Ind. Eng. Chem. Res..

[44] Mathias P.M. (1983). A Versatile Phase Equilibrium Equation of State. Ind. Eng. Chem. Process Des. Dev..

[45] Stryjek R., Vera J.H. (1986). PRSV: An Improved Peng-Robinson Equation of State for Pure Compounds and Mixtures. Can. J. Chem. Eng..

[46] Peneloux A., Rauzy E., Freze R. (1982). A Consistent Correction for Redlich-Kwong-Soave Volumes. Fluid Phase Equilib..

[47] Wertheim M.S. (1984). Fluids with highly directional attractive forces: I. Statistical thermodynamics. J. Stat. Phys..

[48] Wertheim M.S. (1986). Fluids with highly directional attractive forces: IV. Equilibrium polymerization. J. Stat. Phys..

[49] Müller E.A., Gubbins K.E. (2001). Molecular-Based Equations of State for Associating Fluids: A Review of SAFT and Related Approaches. Ind. Eng. Chem. Res..

[50] Chapman W.G., Gubbins K.E., Jackson G., Radosz M. (1989). SAFT Equationof- State Solution Model for Associating Fluids. Fluid Phase Equilib..

[51] Chapman W.G., Gubbins K.E., Jackson G., Radosz M. (1990). New Reference Equation of State for Associating Liquids. Ind. Eng. Chem. Res..

[52] Gross J., Sadowski G. (2002). Application of the Perturbed-Chain SAFT Equation of State to Associating Systems. Ind. Eng. Chem. Res..

[53] Diamantonis N.I., Boulougouris G.C., Mansoor E., Tsangaris D.M., Economou I.G. (2013). Evaluation of Cubic, SAFT, and PC-SAFT Equations of State for the Vapor–Liquid Equilibrium Modeling of CO_2_ Mixtures with Other Gases. Ind. Eng. Chem. Res..

[54] Velasquez J.A., Hernandez J.P., Forero L.A., Cardona L.F. (2022). Prediction of phase equilibria, density, speed of sound and viscosity of 2-alkoxyethanols mixtures: A comparison study between SAFT type EoSs and a modified PR EoS. Fluid Phase Equilib..

[55] Pliego J.R. (2016). Building the liquid-vapour equilibrium curve through a cubic equation of state: Use of excel for teaching physical-chemistry. Quim. Nova.

[56] Kontogeorgis G.M., Privat R., Jaubert J.-N. (2019). Taking Another Look at the van der Waals Equation of State—Almost 150 Years Later. J. Chem. Eng. Data.

[57] Kamesh R., Kumari A., Rani K.Y. (2021). Measurements, Correlations, and Modified UNIFAC Predictions of Isobaric Vapor-Liquid Equilibrium Data for the Binary System of Dimethyl Carbonate plus Anisole at Different Pressures. J. Chem. Eng. Data.

[58] Sarkoohaki B., Almasi M., Karimkhani M. (2019). Theoretical and experimental study of physicochemical behavior of binary mixtures: SAFT and PC-SAFT models. J. Chem. Sci..

[59] Fattahi M., Iloukhani H. (2010). Excess molar volume, viscosity, and refractive index study for the ternary mixture {2-methyl-2-butanol + tetrahydrofuran + propylamine} at different temperatures. Application of the ERAS-model and Peng–Robinson–Stryjek–Vera equation of state. J. Chem. Thermodyn..

[60] Martínez-López J.F., Schneider S., Salavera D., Mainar A.M., Urieta J.S., Pardo J.I. (2016). Molar heat capacities of the mixture {1,8-cineole + ethanol} at several temperatures and atmospheric pressure. J. Chem. Thermodyn..

[61] Torcal M., Langa E., Pardo J.I., Mainar A.M., Urieta J.S. (2016). Isobaric VLE of the mixture {1,8-cineole + ethanol}. EOS analysis and COSMO-RS modeling. J. Chem. Thermodyn..

[62] Garriga R., Sánchez F., Pérez P., Gracia M. (1997). Isothermal vapour-liquid equilibrium at eight temperatures and excess functions at 298.15 K of di-n-propylether with 1-propanol or 2-propanol. Fluid Phase Equilib..

[63] Garriga R., Pérez P., Gracia M. (1997). Vapour Pressures at Eight Temperatures of Mixtures of Di-n-Propylether + Ethanol or 1-ButanolThermodynamic Description of Mixtures of Di-n-Propylether + Alkanol According to the ERAS Model. Ber. Bunsen-Ges. Phys. Chem..

[64] Thermodynamics Research Center (1966). TRC-Thermodynamic Tables-Non-Hydrocarbons.

[65] Ambrose D., Ghiassee N.B. (1987). Vapour pressures and critical temperatures of some alkanoic acids: C1 to C10. J. Chem. Thermodynamic..

[66] Stull D.R. (1947). Vapour Pressure or Pure Substances. Organic Compound. Ind. Eng. Chem..

[67] Guetachew T., Mokbel I., Batiu I., Cisse Z., Jose J. (1999). Vapor pressures and sublimation pressures of eight constituets of essential oils at pressures in the range from 0.3 to 83,000 Pa. ELDATA Int. Electron. J. Phys. Chem. Dat..

[68] Tsonopoulos C. (1974). Empirical Correlation of Second Virial Coeficients. AIChE J..

[69] Erickson W.D., Roach J.F. (1965). Composition and Thermodynamic Properties of Reacting Gas Mixtures under High Pressures Using the Lewis and Randall Rule.

[70] Barker J.A. (1953). Determination of Activity Coefficients from Total Pressure Measurements. Aust. J. Chem..

[71] Wilson G.M. (1964). Vapor-Liquid Equilibrium. 11. A New Expression for the Excess Free Energy of Mixing. J. Am. Chem. Soc..

[72] Rowlinson J.S., Swinton F.L. (1982). Excess thermodynamic functions. Liquids and Liquid Mixtures.

[73] Le Fevre R.J.W., Sundaram A., Pierens R.K. (1963). Molecular Polarisability: The Anisotropy of the Carbon-Oxygen Link. J. Chem. Soc..

[74] Mc Clellan A.L. (1974). Tables of Experimental Dipole Moments.

[75] Meyer L., Büchner A. (1932). The dipolar moment of the n-propyl ether. Physik. Z..

[76] Mc Clellan A.L. (1963). Tables of Experimental Dipole Moments.

[77] Iñarrea J., Valero J., Pérez P., Gracia M., Gutiérrez Losa C. (1988). $H_{m}^{E}$
 and 
$V_{m}^{E}$
 of some (butanone or dipropylether + an alkanol) mixtures. J. Chem. Thermodyn..

[78] Brown I., Fock W., Smith F. (1969). The thermodynamics properties of solutions of normal and branched alcohols in benzene and n-hexane. J. Chem. Thermodyn..

[79] Christensen C., Gmehling J., Rasmussen P., Weidlich U. (1984). Part 1: Heats of Mixing Data Collection. DECHEMA Chemistry Data Series.

[80] Barraza R., Edwards J. (1981). Thermodynamics of the isopropanol/n-hexane and isopropanol/n-heptane systems. Part III. Gas/liquid equilibrium. Monatsh. Chem..

[81] Kashyap P., Rani M., Gahlyan S., Tiwari D.P., Maken S. (2018). Volumetric, acoustic and optical properties of binary mixtures of 2-propanol with n-alkanes (C 6–C 10) from 293.15 K to 303.15 K. J. Mol. Liq..

[82] Letcher T.M., Govender U.P. (1995). CH_4_O and C_4_H_8_O. Primary linear-alkyl monoalcohols + Cyclic monoethers: Heat of Mixing and Solution. J. Chem. Eng. Data.

[83] González J.A., de la Fuente I.G., Cobos J.C. (1996). Disquac analysis of binary-liquid organic mixtures containing cyclic or linear alkanols and cycloalkanes or n-alkanes. Thermochim. Acta.

[84] Lee C., Yang W., Parr R.G. (1988). Development of the Colle-Salvetti correlation-energy formula into a functional of the electron density. Phys. Rev. B.

[85] Becke A.D. (1993). A new mixing of Hartree–Fock and local density-functional theories. J. Chem. Phys..

[86] Becke A.D. (1993). Density functional thermochemistry. III. The role of exact exchange. J. Chem. Phys..

[87] Noury S., Krokidis X., Fuster F., Silvi B. (1999). Computational tools for the electron localization function topological analysis. Comput. Chem..

[88] Patel P., Bhalodia J., Sharma S.S., Chandra P. (2016). Refractive index, speed of sound, FT-IR and computational study of intermolecular interaction between binary mixtures of 1,8-cineole with o-, m- and p-cresol at 303.15, 308.15 and 313.15 K. J. Mol. Liq..

[89] Jimenez J., Valero J., Gracia M., Gutiérrez Losa C. (1988). Hm^E^ of (an n-alkane+ a butanol isomer). J. Chem. Thermodyn..

[90] Pfohl O., Petkov S., Brunner G. (2000). PE 2000. A Poweful Tool to Correlate Phase Equilibria.

[91] Poling B.E., Prausnitz J.M., O’Connell J.P. (2007). The Properties of Gases and Liquids.

[92] Marsh K.N. (1968). Thermodynamics of Octamethylcyclotetrasiloxane Mixtures. Trans. Faraday Soc..

[93] Garriga R., Martínez S., Pérez P., Gracia M. (2001). Isothermal (vapour + liquid) equilibrium at several temperatures of (1-chlorobutane + 1-butanol, or 2-methyl-2-propanol). J. Chem. Thermodyn..

[94] Ambrose D. (1977). Recommended reference materialsfor realization of physicochemical properties. IUPAC Pure Appl. Chem..

[95] Smith A., Menzies A.W.C. (1910). Studiesin Vapor Pressure: III. A Static Method for Determining the Vapor Pressures of Solids and Liquids. J. Am. Chem. Soc..

[96] Head A.J., Sabbah R., Marsh K.N. (1987). Enthalpy. Recommended Reference Materials for the Realization of Physicochemical Properties.

[97] Lafuente C., Artigas H., Lopez M.C., Royo F.M., Urieta J.S. (2001). Excess Molar Enthalpies for Isomeric Chlorobutanes with Isomeric Butanols. Phys. Chem. Liq..

[98] Zafarani-Moattar M.T., Shekaaria H. (2007). Density and speed of sound of lithium bromide with organic solvents: Measurement and correlation. J. Chem. Thermodyn..

[99] Haase R., Tillmann W. (1994). Properties of the liquid-system water plus 2-propanol. Z. Phys. Chem..

[100] Dubey G.P., Sharma M. (2009). Studies of mixing properties of binary systems of 2-propanol with hexadecane and squalane at T = (298.15, 303.15, and 308.15). J. Chem. Thermodyn..

[101] Aminabhavi T.M., Aralaguppi M.I., Harogoppad S.B., Balundgi R.H. (1993). Densities, viscosities, refractive indices, and speeds of sound for methyl acetoacetate + aliphatic alcohols (C1–C8). J. Chem. Eng. Data.

[102] Barata P.A., Serrano M.L. (1994). Densities and Viscosities of Thymol + 1,8-Cineole. J. Chem. Eng. Data.

